# Survival of white-tailed deer fawns in central Iowa

**DOI:** 10.1371/journal.pone.0229242

**Published:** 2020-03-03

**Authors:** Patrick G. McGovern, Stephen J. Dinsmore, Julie A. Blanchong

**Affiliations:** Department of Natural Resource Ecology and Management, Iowa State University, Ames, Iowa, United States of America; Universita degli Studi di Sassari, ITALY

## Abstract

Understanding demographic parameters such as survival is important for scientifically sound wildlife management. Survival can vary by region, sex, age-class, habitat, and other factors. White-tailed deer fawn survival is highly variable across the species’ range. While recent studies have investigated fawn survival in several Midwestern states, there have been no published estimates from Iowa for 30 years. We radio-collared 48 fawns in central Iowa from 2015–2017 to estimate survival, home range size, and habitat composition and identity causes of mortality. Estimated fawn survival (± SE) was similar to other Midwest studies at 30 (0.78 ± 0.07)) and 60 days (0.69 ± 0.08), but considerably lower at 7 months (0.31 ± 0.02). Survival was positively associated with woodland habitat through 30 and 60 days, but not related to habitat at 7 months. Female fawns avoided agricultural habitat in their home ranges. Fawn 95% kernel density home ranges were smaller than in other studies in the Midwest (21.22 ± 2.74 ha at 30 days, 25.47 ± 2.87 ha at 60 days, and 30.59 ± 2.37 ha at 7 months). The large amount of woodland and grassland (>90%) in our study area meant that fawns did not have to travel far to find suitable cover, which may explain their small home ranges. We recorded 21 mortalities, the leading cause of which was disease (n = 9; 56% epizootic hemorrhagic disease [EHD]) followed by suspected predation (4) and harvest (3). The mortality associated with an outbreak of EHD in 2016, all of which occurred after 60 days post-capture, is the most likely explanation for our low survival estimate at 7 months. While predation, usually early in life, is the leading cause of mortality in most studies, sporadic diseases like EHD can be a major source of mortality in older fawns in some years.

## Introduction

Understanding demographic parameters such as survival is important for scientifically sound management of wildlife populations. Survival rates can be estimated for individuals grouped by factors such as region [1], sex [2], or age-class [3]. Differences in age-specific survival can be the result of variation in predation risk, foraging efficiency, or disease susceptibility [4, 5]. Neonatal ungulates have the lowest survival rates of any age class and are more susceptible than adults to several sources of mortality including predation, disease, and malnutrition in part due to their limited mobility, underdeveloped immune systems, and dependence upon their mother for nutrition [5–7]. How individuals within a population utilize the landscape may affect their survival by altering exposure to particular sources of mortality [8, 9]. For instance, neonates in areas with a high concentration of available resources often have small home range sizes, which in turn means individuals move shorter distances or less often [10]. Increased movement distance and frequency, on the other hand, can increase exposure to predators [11, 12]. Selection for habitat types that provide hiding cover may decrease predation risk among neonates that rely on concealment while avoidance of roads will reduce the risk of collision with vehicles [13–15].

As with other ungulates, neonatal white-tailed deer (*Odocoileus virginianus*) suffer higher rates of mortality than yearlings or adults [6]. The primary source of fawn mortality documented in most studies is predation [see 16]. Other common sources of mortality include starvation [17] and harvest [14, 18]. Fawn survival rates are highly variable across studies and are generally higher in the Midwest than in other portions of the white-tailed deer range in North America [see 16]. Agricultural, particularly row-crop, land use in these states provides ample food resources for adult deer, likely increasing their condition during winter and gestation [14]. The nutritional condition of adult females may have knock-on effects to fawn survival by increasing birth mass or reducing abandonment rates [19]. Fawn body size and birth mass can positively influence survival estimates [20]. Many studies in Midwestern states also took place in areas that lack the diverse communities and densities of predators found in more forested regions of the northern or eastern United States [21–23].

While fawn survival has been investigated in several Midwestern states recently [e.g., 15, 17, 23, 24], there have been no published studies from Iowa for several decades [25]. In the years since, the Iowa white-tailed deer population has peaked and management strategies have shifted from promoting growth to maintaining steady herd numbers through harvest [26]. We studied fawn survival in central Iowa. Our objectives were to 1) estimate fawn survival and identify habitat variables associated with survival, 2) estimate fawn home range size, 3) evaluate habitat selection within home ranges, and 4) attempt to document causes of mortality.

## Materials and methods

### Study area

Our study was conducted from 2015–2017 in a 10 km^2^ area in Boone County in central Iowa, USA (study area centroid: 41.973189, -93.891365). We worked on private (35%) and public (65%) lands along the Des Moines River. Public lands included Ledges State Park (5 km^2^) and McCoy Wildlife Management Area (1.75 km^2^). Steep sandstone ridges and ravines provided topographical variation with a maximum elevation change of 75 m. Our study area was 80% woodland, 11% grassland, 6% agriculture, 2% developed (structures and roads), and 1% water/wetland [calculated from 27]. Common tree species included white oak (*Quercus alba*), red oak (*Q*. *rubra*), American linden (*Tilia americana*), and black walnut (*Juglans nigra*). Herbaceous cover was primarily Virginia creeper (*Parthenocissus quinquefolia*), sedges (*Carex* spp.), *Sanicula* spp., American hogpeanut (*Amphicarpaea bracteata*), and pointed-leaved tick trefoil (*Desmodium glutinosum*). Prairie grasses included big bluestem (*Andropogon gerardii)*, little bluestem (*Schizachyrium scoparius*), switchgrass (*Panicum virgatum*), and Canada wild rye (*Elymus canadensis*) [28]. Potential predators of fawns in our study area included coyotes (*Canis latrans*), domestic dogs (*Canis familiaris)* and possibly bobcats (*Lynx rufus*); [29]). The deer population in Iowa was considered stable by the Iowa Department of Natural Resources [26, 30, 31]. As was true across Iowa, harvest was used in our study area to manage deer population density, specifically to prevent the deer population from becoming overabundant [26, 30, 31]. From 1 October to early January each year, McCoy Wildlife Management Area was open to all forms of legal deer hunting and Ledges State Park was open to antlerless-only archery hunts. For private land, availability of the property for hunting was at the discretion of the owner.

### Search and capture

We captured neonatal fawns during late May and early June 2015–2017. We grid-searched woodland and grassland habitat on foot with crews of 2–8 searchers to locate bedded fawns. We also opportunistically located fawns by observing doe behavioral cues [32, 33] and from sightings reported by the public. We captured fawns by hand at their bed site or after a brief (<10 m) chase. We blindfolded fawns to reduce external stimuli and, if we captured them at a bed site, moved them ≤10 m from the bed site to avoid disturbing vegetation. We fit each fawn with an expandable, breakaway VHF radio collar (model M4210, Advanced Telemetry Systems [ATS], Isanti, MN, USA) programmed with a 4- or 12-hour mortality switch [34]. We wore unscented nitrile gloves for all handling and gently rubbed fawns with native vegetation before release to minimize human scent transfer. We handled fawns for ≤15 minutes (mean = 7.8, SD = 3.1) to minimize the risk of handling-related abandonment. After processing, we returned fawns to their bed sites and searched around the capture site to locate possible siblings. The Iowa State University Institutional Animal Care and Use Committee approved this study (Protocol No. 2-15-7954-W). We collected fawns under IDNR Scientific Collector Permit No. SC871 and in accordance with the guidelines of the American Society of Mammalogists [35].

### Monitoring

We monitored fawns using radio telemetry 1 to 4 times per day from capture through 31 August and twice per week until 31 December of their capture year. If we could not detect a radio signal from a collar using a handheld Yagi antenna or vehicle-mounted omnidirectional antenna and receiver (model R410, ATS) during a monitoring period, we expanded the search area and attempted again during the following monitoring period. If a signal was not detected after two consecutive days, we assumed the fawn had left the study area or the collar had failed, and we censored it from survival analysis at its last known signal date.

We located fawns by radio telemetry using a handheld Yagi antenna and receiver. We used a rotating 4-hour block monitoring schedule (e.g., 0600–1000 h, 1000–1400 h, 1400–1800 h, 1800–2200 h) through 31 August to stratify fawn locations throughout the day. We visually confirmed the location of fawns tracked with telemetry at least once per week from capture to 31 August and recorded their location to within ≤10 m using a handheld GPS. After 31 August, we visually confirmed fawn locations on an *ad hoc* basis. When we did not visually locate fawns, we estimated fawn locations through triangulation by taking a set of ≥3 bearings within 45 minutes using a handheld compass. We estimated locations using the maximum likelihood estimator in program LOAS (version 4.0.3.8, Ecological Software Solutions LLC, Hegymagas, Hungary; [36]). We only included locations with a 95% χ^2^ error ellipse ≤2 ha in our analysis [37].

### Survival estimates

We estimated survival for fawns in three time intervals: 0–30 days, 0–60 days, and 0 days– 7 months post-capture. The intervals were determined for each individual fawn based on its date of capture. These three intervals were selected because of differences in behavior that might affect survival. Specifically, we considered all fawns “hiders” from capture to 30 days post-capture [38]. Because the transition from the “hider” to the more mobile “follower” stage is not a discrete event, we developed a second interval that included 31–60 days post-capture where fawns were in transition from “hiders” to “followers”. For our third interval, we included 61 days– 7 months post-capture and assumed all fawns were no longer “hiders” by day 61. We chose 7 months rather than the more commonly reported 6 months to encompass the majority of the Iowa deer hunting season. To facilitate comparison to other studies we also report survival through 6 months age.

We estimated fawn survival for the time intervals described above using the Known Fate analysis in Program MARK [39]. We used a staggered entry design where individuals entered the analysis on date captured. We left-censored all dropped or lost collars to be as conservative as possible in our estimates. To model the effects of key habitat variables on fawn survival, rather than using home-ranges, we created circular buffers [e.g., 40, 41] around each fawn’s location at each monitoring occasion within an interval and calculated the proportion of woodland and agricultural habitat in ArcGIS (version 10.3, Environmental Systems Research Institute, Inc., Redlands, CA, USA) using a 3 m high-resolution land cover raster layer [27]. Creating circular buffers to quantify habitat rather than using the home range is a commonly used approach when not all animals have sufficient relocations to allow home ranges to be estimated rigorously [e.g., 42, 43]. Because fawns were located multiple times per day through August we arbitrarily selected the location closest to noon each day to create buffers. The radius of the buffer in each of the three intervals was based on mean daily fawn movements calculated across all fawns for that interval. We estimated mean daily fawn movement by averaging the linear distance between locations of individual fawns recorded 12–36 hours apart in ArcGIS. Mean daily fawn movement and associated buffer radius ± standard error (SE) was 120 ± 5.3 m for the 30-day interval, 155 ± 4.1 m for the 60-day interval, and 170 ± 3.8 m for the 7-month interval.

We considered separate candidate model sets for fawn survival in each of the three time intervals. For all intervals, models included constant survival {S(.)}, linear trend {(S(T)}, quadratic trend {S(T^2^)}, weekly survival {S(Weekly)}, and covariate-specific models. For the 0–60 day interval, interval-specific survival {S(30, 60)} was also included, and for the 0–7 month model, survival between non-hunting and hunting seasons {S(Hunt)} and behavioral stage {S(Hider)}were also included. Covariates included year, sex, proportion of woodland habitat, and proportion of agricultural habitat. Our modeling approach included mostly single-effect models because our small sample size of fawns reduced radio-days such that multivariate models would not converge. We did, however, allow linear (T) and quadratic (TT; 7-month only) trends within year to be additive on a year effect. We ranked survival models using Akaike’s Information Criterion (AIC) corrected for small sample size (AIC_c_; [44]) and selected the model with the smallest AIC_c_ as the best model for each interval. We considered models to have no support if they were >2 AIC_c_ from the best model (ΔAIC_c_; [44]). When another single-effect model was within 2 AIC_c_ of the top model, we fit an additive model containing both effects. If the additive model was more supported than the top single-effect model (i.e., more than 2 AIC_c_ units better), we also fit of a model containing an interaction between the two effects. We used the Bayesian Markov chain Monte Carlo estimation tool in Program MARK to aid parameter estimation. All parameter estimates were reported with 95% credible intervals (CI) and slope parameters were considered significant when the 95% CI did not overlap zero.

### Home range estimation and habitat composition

We created home ranges for fawns in the three time intervals: 0–30 days, 0–60 days, and 0 days– 7 months post-capture. A fawn was included in an interval if it had ≥30 locations within the interval of interest [45, 46]. We estimated 95% kernel density home ranges using the least squares cross validation (LSCV) bandwidth estimator in Geospatial Modeling Environment (GME; version 0.7.4, Spatial Ecology LLC). We calculated the area and habitat composition of each home range in ArcGIS using a 3 m high-resolution land cover raster layer [27]. We limited analyses to habitat classes that we believed may have implications for fawn survival: woodland, grassland (which included pasture, prairie, and lawns), agriculture (which included row-crop and all other types), and roads. We fit mixed-effects models to identify explanatory variables associated with the area of a fawn’s home range. Variables included time interval and sex as fixed effects and year as a random effect. We fit additional mixed-effects models to test for differences in home range composition with habitat class as the response variable, sex and time interval as fixed effects, and year as a random effect. We evaluated significance of fixed effects using ANOVA and *t*-tests at α = 0.05.

We assessed habitat selection for male and female fawns separately for each time interval using Manly’s selection ratio [47] in package ‘adehabitat’ in R (R Version 3.3.3, www.r-project.org). We tested third-order habitat selection, where available habitat was unique to each fawn [48]. We quantified available habitat as the proportion of each habitat class present within a fawn’s home range and we quantified habitat use as the total number of locations of a fawn in each habitat class. Manly’s selection ratio provides a mean ‘weight’ value for each habitat class [47]. Values >1 indicate selection for a habitat class and values <1 indicate selection against a habitat class. We considered selection significant when the 95% confidence interval for a value did not include 1. We omitted the road habitat class from the habitat selection analysis because our definition of road use required fawns to be physically located on a road, which rarely occurred.

### Cause of mortality

When a collar broadcast a mortality signal, we located and examined the collar, the carcass, and the surrounding area for indications of cause of death including blood, wounds, and tracks. We classified the most likely cause of mortality as disease, harvest, starvation, suspected predation/scavenging, vehicle collision, or unknown based on the condition of the remains and surrounding area. We submitted intact carcasses to the Veterinary Diagnostic Laboratory at Iowa State University for gross necropsy. If we found a collar and no remains or indication of mortality (hair, blood, drag marks, etc.), we classified the fate as unknown.

## Results

### Survival estimates

**Fig 1:**
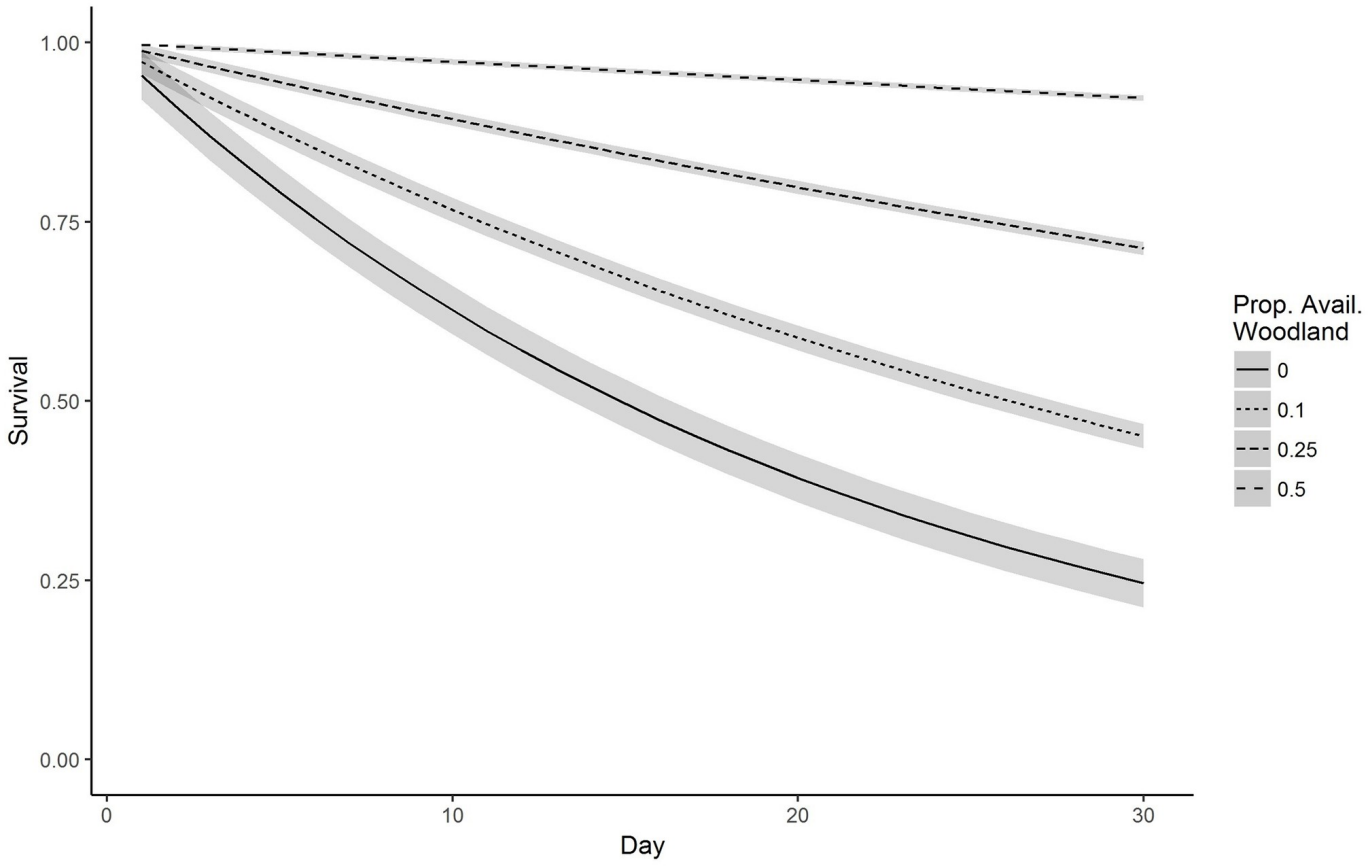
Predicted survival of white-tailed deer fawns to 30 days post-capture based on the top model {S(Wood)} for a range of proportions of woodland available within 120 m of fawn locations, Boone County, Iowa, USA, 2015–2017. Shaded regions indicate 95% credible intervals.

**Fig 2:**
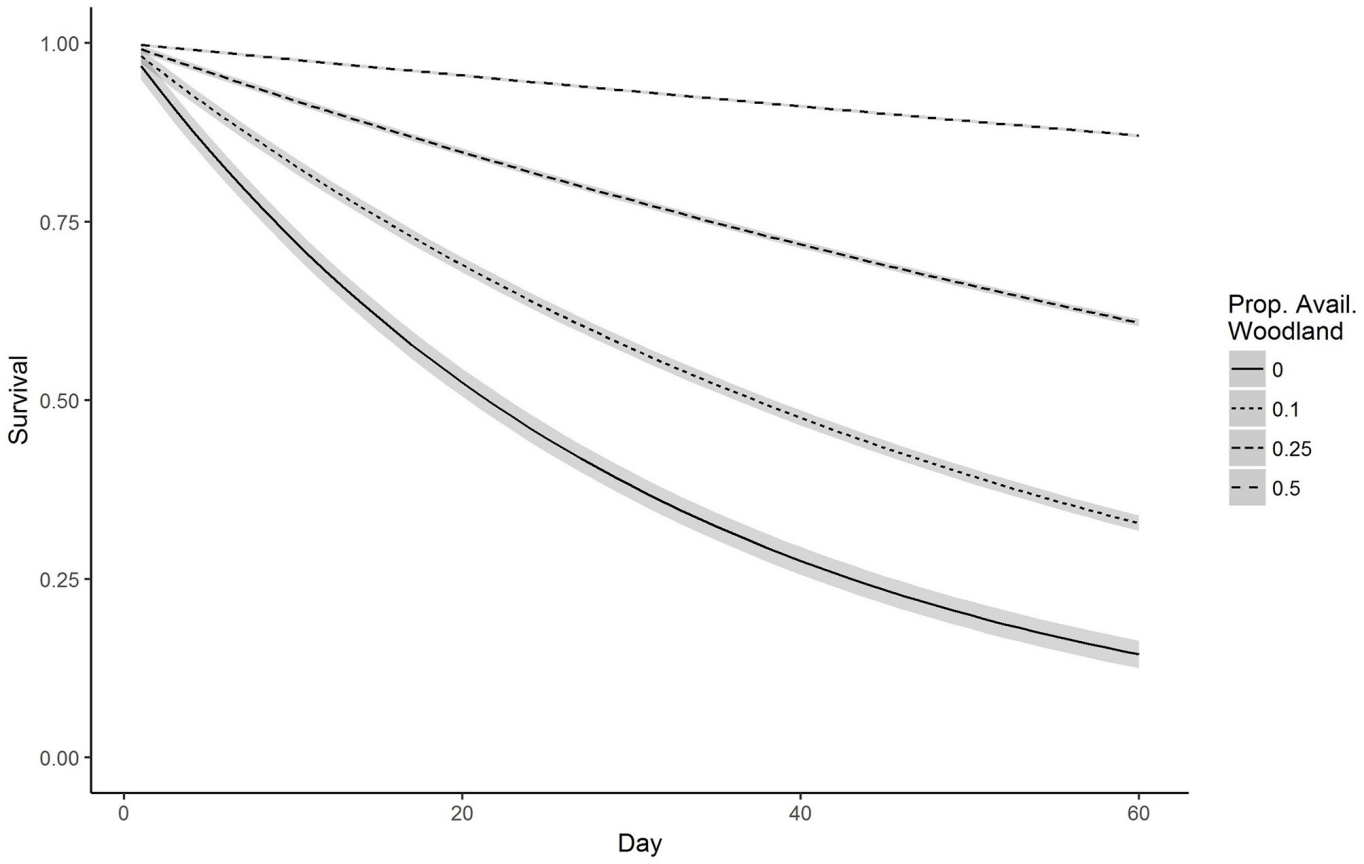
Predicted survival of white-tailed deer fawns to 60 days post-capture based the top model {S(Wood)} for a range of proportions of woodland available within 155 m of fawn locations, Boone County, Iowa, USA, 2015–2017. Shaded regions indicate 95% credible intervals.

We captured and radio-collared 48 fawns (22 male, 26 female) from 2015–2017 (12 in 2015, 24 in 2016, 12 in 2017), including two pairs that appeared to be twins based on timing and proximity of capture. While the findings from twins may be confounded we elected to include both fawns for the sake of sample size. Mean date of capture was 27 May (range: 15 May– 17 June). Our top model for survival through 30 days post-capture included a statistically significant positive effect of proportion of woodland (β = 3.81, 95% CI 2.08, 5.71; Table 1 and Fig 1). Estimated 30-day survival (±SE) from mean date of capture was 0.78 ± 0.07. The only other single-effect model within 2 ΔAIC_c_ of proportion of woodland was the proportion of agriculture model (Table 1). Because woodland and agriculture were correlated, the two effects could not be included in the same model. In addition, proportion of agriculture was not statistically significant (β = 1.54, 95% CI −1.3, 4.53). Our top model for 60-day survival included a statistically significant positive effect of proportion of woodland (β = 5.43, 95% CI 3.43, 7.45; Table 2 and Fig 2). Estimated 60-day survival was 0.69 ± 0.08. No other single-effect model was within 2 ΔAIC_c_ of the top model. For 7-month survival, our top single-effect model was proportion of woodland. Another single-effect model including a quadratic trend was within 2 ΔAIC_c_ of the top model, so we fit an additive model including proportion of woodland and a quadratic trend. This model was a better fit than proportion of woodland alone so we then fit a model with an interaction between proportion of woodland and a quadratic trend. This model was our top model although no effects were statistically significant (Table 3). Estimated 7-month survival was 0.31 ± 0.02. For comparison to other studies, 6-month survival was 0.37 ± 0.02. Models including sex or year as a main effect were not competitive (ΔAIC_c_ > 2) in any time intervals.

**Table 1:** Candidate model set for survival of white-tailed deer fawns through 30 days post-capture, Boone County, Iowa, USA, 2015–2017.

Model	Delta AICc^g^	AICc Weights	Model Likelihood	Num. Par.^h^
**{S(Wood)}**^a^	0.00	0.61	1.00	2
**{S(Ag)}**^b^	0.89	0.39	0.65	2
**{S(T)}**^c^	19.14	0.00	0.00	2
**{S(Weekly)}**^d^	19.30	0.00	0.00	8
**{S(.)}**^e^	20.31	0.00	0.00	1
**{S(Sex)}**	20.38	0.00	0.00	2
**{S(T**^**2**^**)}**^f^	20.86	0.00	0.00	3
**{S(Year+T)}**	22.66	0.00	0.00	4
**{S(Year)}**	23.89	0.00	0.00	3

^a^Wood: proportion of woodland habitat within 120 m of fawn locations

^b^Ag: proportion of agriculture habitat within 120 m of fawn locations

^c^T: linear trend

^d^Weekly: week of interval

^e^(.): constant trend

^f^T^2^: quadratic trend

^g^Delta AIC_c_: difference in Akaike’s Information Criterion corrected for small sample size between model and top model

^h^Num. Par.: number of parameters in model

**Table 2:** Candidate model set for survival of white-tailed deer fawns through 60 days post-capture, Boone County, Iowa, USA, 2015–2017.

Model	Delta AICc^h^	AICc Weights	Model Likelihood	Num. Par.^i^
**{S(Wood)}**^a^	0.00	1.00	1.00	2
**{S(Ag)}**^b^	26.16	0.00	0.00	2
**{S(T)}**^c^	47.87	0.00	0.00	2
**{S(T**^**2**^**)}**^d^	49.79	0.00	0.00	3
**{S(Year + T)}**	50.21	0.00	0.00	4
**{S(30, 60)}**^e^	51.41	0.00	0.00	2
**{S(Sex)}**	52.11	0.00	0.00	2
**{S(Weekly)}**^f^	52.67	0.00	0.00	12
**{S(.)}**^g^	52.79	0.00	0.00	1
**{S(Year)}**	55.04	0.00	0.00	3

^a^Wood: proportion of woodland habitat within 155 m of fawn locations

^b^Ag: proportion of agriculture habitat within 155 m of fawn locations

^c^T: linear trend

^d^T^2^: quadratic trend

^e^30, 60: interval-specific model

^f^Weekly: week of interval

^g^(.): constant trend

^h^Delta AIC_c_: difference in Akaike’s Information Criterion corrected for small sample size between model and top model

^i^Num. Par.: number of parameters in model

**Table 3:** Candidate model set for survival of white-tailed deer fawns through 7 months post-capture, Boone County, Iowa, USA, 2015–2017.

Model	Delta AICc^i^	AICc Weights	Model Likelihood	Num. Par.^j^
{S(Wood * T^**2**^**)}**^a^^,^^b^	0.00	0.95	1.00	6
**{S(Wood + T**^**2**^**)}**	7.01	0.03	0.03	4
**{S(Wood)}**	9.30	0.01	0.01	2
**{S(T**^**2**^**)}**	9.90	0.01	0.01	3
**{S(.)}**^c^	13.38	0.00	0.00	1
**{S(Hider)}**^d^	15.26	0.00	0.00	2
**{S(Pre/Post Hunt)}**^e^	15.32	0.00	0.00	2
**{S(T)}**^f^	15.35	0.00	0.00	2
**{S(Ag)}**^g^	15.38	0.00	0.00	2
**{S(Sex)}**	15.38	0.00	0.00	2
**{S(Year)}**	16.77	0.00	0.00	3
**{S(Year + T)}**	18.76	0.00	0.00	4
**{S(Weekly)}**^h^	43.28	0.00	0.00	34

^a^Wood: proportion of woodland habitat within 170 m of fawn locations

^b^T^2^: quadratic trend

^c^(.): constant trend

^d^Hider: behavioral stage

^e^Pre/Post Hunt: hunting season status

^f^T: linear trend

^g^Ag: proportion of agriculture habitat within 170 m of fawn locations

^h^Weekly: week of interval

^i^Delta AIC_c_: difference in Akaike’s Information Criterion corrected for small sample size between model and top model

^j^Num. Par.: number of parameters in model

#### Home range estimation and habitat composition

We recorded 4,280 locations (range: 1–168 locations per fawn) between capture and 31 December each year across the 3 years of the study. We estimated 95% kernel density home ranges for 36 fawns from 0–30 days post-capture (mean: 31 locations per fawn), 33 fawns from 0–60 days post-capture (73 locations), and 29 fawns from 0 days– 7 months post-capture (128 locations). Home ranges averaged 21.22 ± 2.74 SE ha for the 0–30 day interval, 25.47 ± 2.87 ha for the 0–60 day interval and 30.59 ± 2.37 ha for the 0 day–7 month interval. Home range size was statistically different among intervals (*F*_2,90_ = 4.16, *P* < 0.05), with 7-month home ranges larger than 30-day ranges (*t*_92_ = 2.88, *P* < 0.01). Males had larger home ranges than females across all intervals (*t*_92_ = 2.66, *P* < 0.01; Table 4).

**Table 4:** Average home range area (in ha) of white-tailed deer fawns by sex and time interval, Boone County, Iowa, USA, 2015–2017.

	Area (SE)	
Interval	Males	Females
**30 days**	28.7 (5.62)	15.8 (1.79)
**60 days**	33.1 (6.24)	21.1 (2.42)
**7 months**	33.0 (4.76)	29.3 (2.66)

Woodland comprised >60% of home range habitat in all time intervals, followed by grassland (>15%), agriculture (<10%), and roads (<5%; Table 5). Woodland and grassland, woodland and agriculture, and woodland and roads were negatively correlated (Spearman’s Rank correlation coefficient (r_s_) = −0.90 to −0.75, all *P* < 0.001). Grassland and roads, grassland and agriculture, and agriculture and roads were positively correlated (r_s_ = 0.58 to 0.75, all *P* < 0.001). Given these correlations, we ran four mixed-effects models with each habitat class of interest as the response variable, year as a random effect, and interval and sex as fixed effects to test for differences in home range habitat composition among time intervals or between male and female fawns. The average amount of woodland in home ranges was not statistically significantly different among time intervals (*F*_2,92_ = 0.22, *P* > 0.05; Table 5) or between males and females (*t*_92_ = −1.01, *P* > 0.05; Table 6). The average amount of grassland in home ranges was not statistically significantly different among time intervals (*F*_2,92_ = 0.14, *P* > 0.05; Table 5) or between males and females (*t*_92_ = 0.86, *P* > 0.05; Table 6). The average amount of agriculture in home ranges was not statistically significantly different among time intervals (*F*_2,92_ = 0.31, *P* > 0.05; Table 5) or between males and females (*t*_92_ = 0.79, *P* > 0.05; Table 6). The average amount of road in fawn home ranges was not statistically significantly different among intervals (*F*_2,92_ = 0.13, *P* > 0.05; Table 5) or between males and females (*t*_92_ = 0.23, *P* > 0.05; Table 6).

**Table 5:** Average habitat composition of white-tailed deer fawn home ranges for time intervals of 0–30 days post-capture, 0–60 days post-capture, and 0 days–7 months post-capture, Boone County, Iowa, USA, 2015–2017.

	Percent Composition (SE)		
Habitat Class	30-day	60-day	7-month
**Woodland**	70.73 (4.22)	67.30 (4.33)	70.31 (3.97)
**Grassland**	17.16 (2.51)	19.08 (2.62)	19.12 (2.60)
**Agriculture**	8.21 (2.24)	9.47 (2.71)	6.74 (2.00)
**Roads**	3.90 (0.47)	4.15 (0.47)	3.83 (0.43)

**Table 6:** Habitat composition of male and female white-tailed deer fawn home ranges averaged across intervals of 0–30 days post-capture, 0–60 days post-capture, and 0 days–7 months post-capture, Boone County, Iowa, USA, 2015–2017.

	Percent Composition (SE)	
Habitat Class	Males	Females
**Woodland**	65.86 (4.04)	71.63 (2.99)
**Grassland**	20.50 (2.73)	17.10 (1.70)
**Agriculture**	9.60 (2.53)	7.35 (1.55)
**Roads**	4.04 (0.36)	3.92 (0.37)

**Fig 3:**
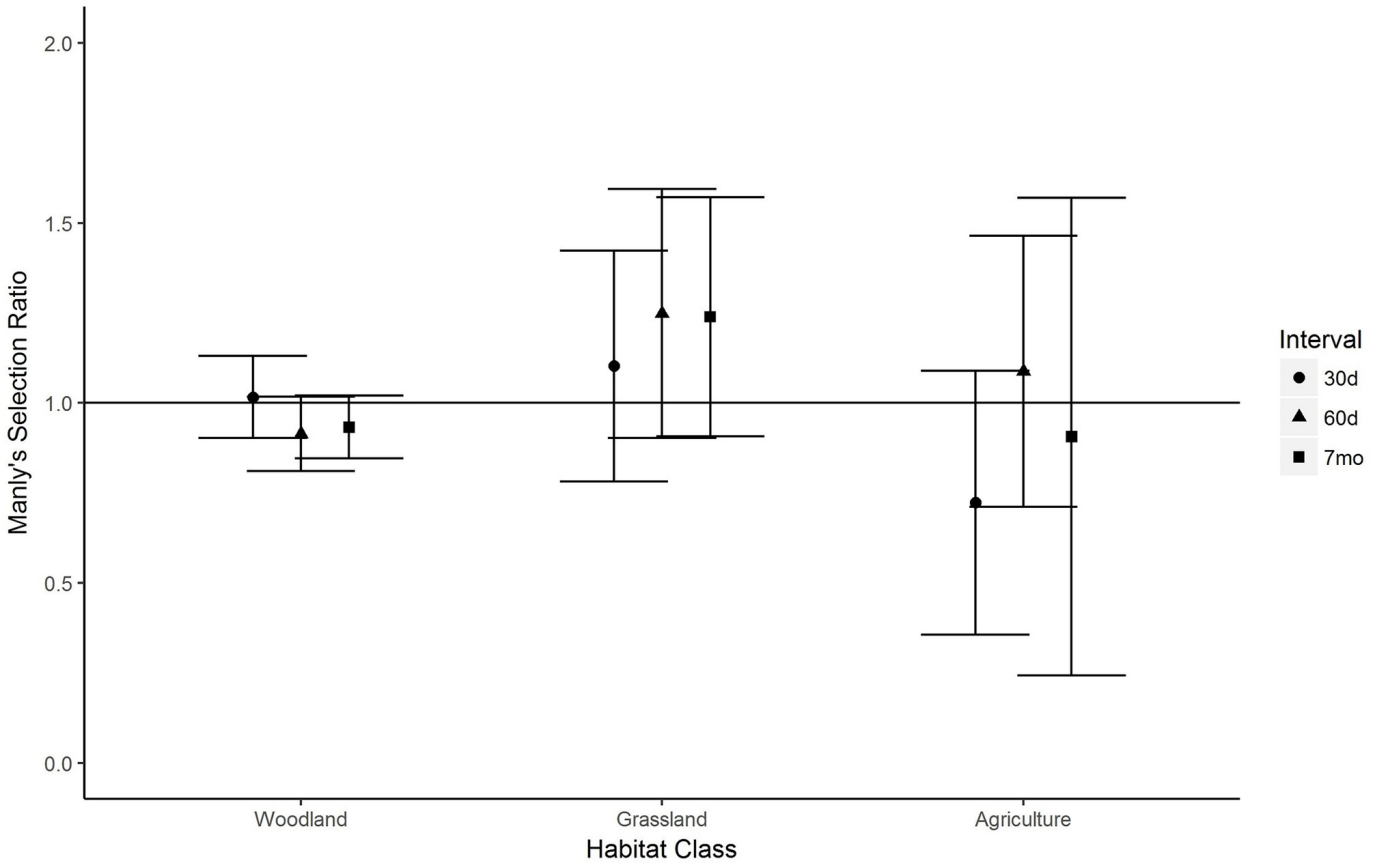
Habitat selection by male white-tailed deer fawns over intervals of 0–30 days post-capture, 0–60 days post-capture, and 0 days–7 months post-capture, Boone County, Iowa, USA, 2015–2017. Selection ratios >1 indicate selection for a habitat class, while ratios <1 indicate selection against a habitat class. Error bars represent 95% confidence intervals.

**Fig 4:**
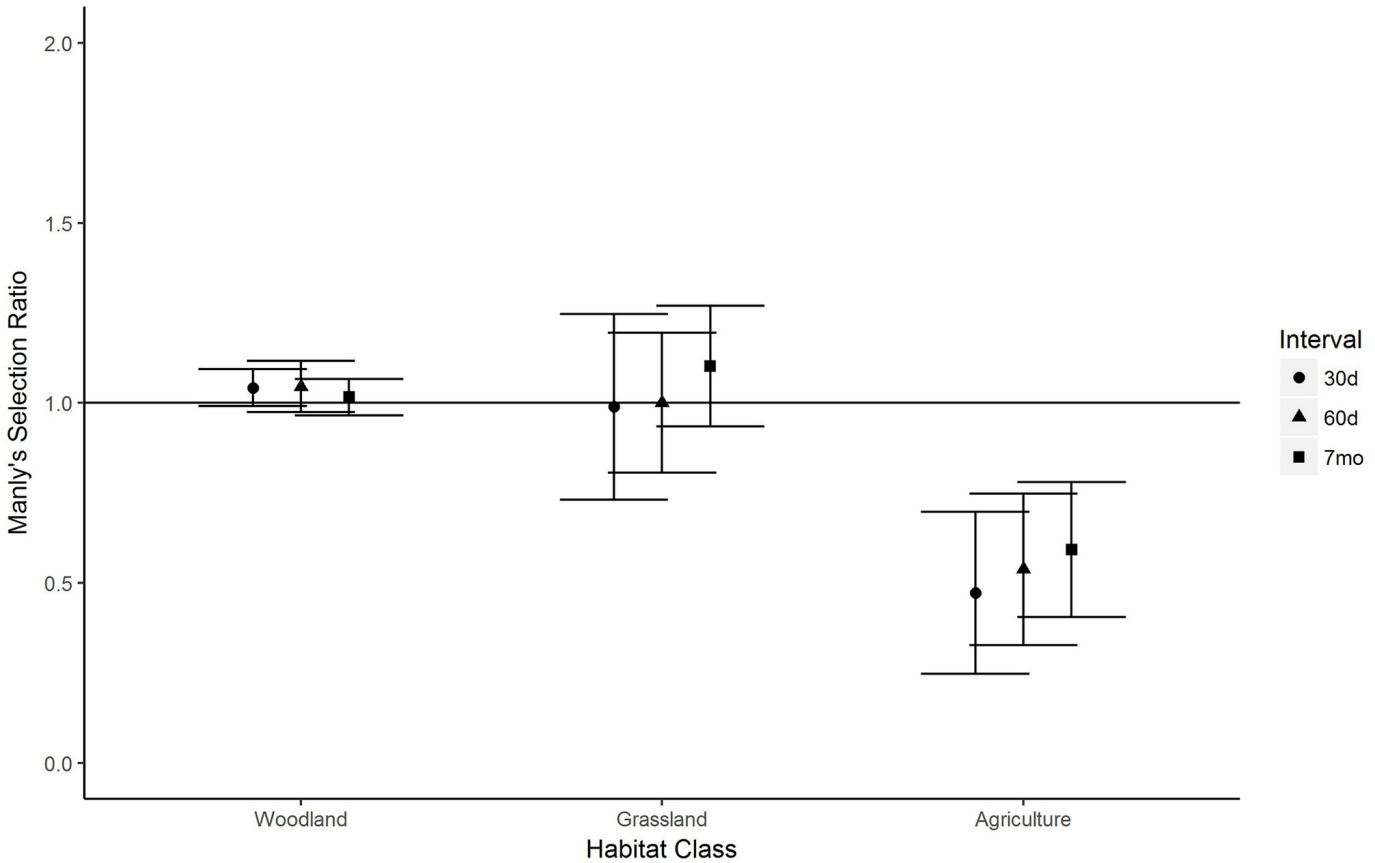
Habitat selection by female white-tailed deer fawns over intervals of 0–30 days post-capture, 0–60 days post-capture, and 0 days–7 months post-capture, Boone County, Iowa, USA, 2015–2017. Selection ratios >1 indicate selection for a habitat class, while ratios <1 indicate selection against a habitat class. Error bars represent 95% confidence intervals.

Male fawns did not exhibit habitat selection within their 30-day home ranges (χ_19_^2^ = 27.38, *P* > 0.05), but did at 60 days (χ_21_^2^ = 43.61, *P* < 0.01) and 7 months (χ_18_^2^ = 50.81, *P* < 0.001). However, individual habitat selection ratios were not significant in any interval (Fig 3). Female fawns did not exhibit habitat selection within their 30-day home ranges (χ_23_^2^ = 32.01, *P* > 0.05), but did at 60 days (χ_33_^2^ = 68.00, *P* < 0.001) and 7 months (χ_35_^2^ = 63.45, *P* < 0.01). Female fawns selected against agricultural habitat in all time intervals (Fig 4).

### Cause of mortality

We recorded 21 mortalities (9 male, 12 female) between capture and 31 December of the capture year (4 in 2015, 12 in 2016, 5 in 2017). Eleven mortalities were found on public land and 10 on private land. We recorded 8 mortalities within 30 days of capture, 2 mortalities in the period 31–60 days post-capture, and 11 mortalities in the period 61 days-7 months post-capture. We were not confident in our ability to identify a specific predator associated with a mortality or to discriminate it from scavenging and therefore grouped mortalities where there was evidence that the fawn was at least partially consumed. The primary source of mortality was disease (Table 7), including epizootic hemorrhagic disease [EHD] (5), enteritis (3), and sepsis (1). We submitted samples from the first suspected EHD mortality to the U.S. Department of Agriculture National Veterinary Services Laboratories to confirm the gross necropsy diagnosis of EHD by polymerase chain reaction (PCR) test, which identified EHD virus serotype 6. We classified subsequent EHD mortalities solely by necropsy.

**Table 7:** Cause-specific mortality of white-tailed deer fawns, Boone County, Iowa, USA, 2015–2017.

Cause	2015	2016	2017	Total	Percent of Total Mortality
**Disease**	0	8	1	9	43
**Suspected predation**	2	0	2	4	19
**Harvest**	1	2	0	3	14
**Vehicle collision**	0	1	0	1	5
**Starvation**	0	0	1	1	5
**Unknown**	1	1	1	3	14
**All causes**	4	12	5	21	

## Discussion

### Survival estimates

As has been documented in other studies in Midwestern states, we found high 30- and 60-day survival for fawns in central Iowa. Our 30-day fawn survival estimate was comparable to survival estimates from studies in Iowa (0.86; [25]) and Michigan (0.93–0.97; [18]) that used similar capture methods. It is important to note that because we searched for fawns, rather than using vaginal implant transmitters (VITs) that transmit a signal at birth, we may have missed mortality that occurred shortly after birth [49, 50]. As a result, we have likely overestimated survival for the 30-day survival interval. Fawn survival through 60 days in our study was similar to previous estimates from Michigan (0.81; [17]), Iowa (0.85; [25]), and southern Minnesota and South Dakota (0.89; [24]). For both 30- and 60-day survival we found a statistically significant positive relationship with the proportion of woodland habitat. A model including the proportion of agricultural habitat was competitive for 30-day survival, but the parameter estimate was not statistically significant. The majority of habitat in our study area was woodland (>80%). Areas with higher proportions of agriculture have been hypothesized to support higher nutritional condition in lactating females thereby contributing to higher fawn survival [23]. However, in early summer in Iowa (May-June), the row-crop agriculture in our study area offers no cover for fawns. As a result, woodland habitat during this time frame may be more important for survival because it offers cover during the “hider” life stage. Large forest patches similarly positively influenced fawn survival in southern Illinois [42].

Our survival estimates through 6 and 7 months post-capture (0.37, 0.31 respectively) were lower than our 30- and 60-day estimates and lower than findings in comparable Midwestern fawn studies. Fawn survival through 6 months was 0.77 in an earlier study in Iowa [25] and 0.67 in Michigan [17]. A study in Michigan reported 220-day survival estimates of 0.76 and 0.85 in two different years [18]. Unlike the earlier intervals, no parameters in our top model for 7-month survival were statistically significant. This contrasts with a recent meta-analysis of fawn survival studies across North America that found survival for 3- to 6-month-old fawns was positively related to the proportion of agricultural habitat [16]. Our failure to find this relationship may be related to the relatively small proportion of agriculture in our study area. It may also be related to the primary cause of mortality in our study. Unlike most studies, where predation was responsible for the large majority of mortality [see 16], disease, primarily EHD, was the leading cause in our study. There is no evidence that we are aware of to suggest that nutritional condition is related to susceptibility to EHD. The mortality associated with a sporadic outbreak of EHD in 2016, all of which occurred during the third time interval, is the most likely explanation for our overall low survival estimate through 7 months of age and perhaps our failure to identify a relationship between survival and agricultural habitat.

### Home range and habitat composition

Our estimated home ranges were smaller than most fawn home ranges reported for comparable time intervals in the Midwest. Fawn home ranges in Michigan averaged 40.9 ha through 2 months [17] and 62.65 ha through 27 weeks [18]. In South Dakota and Minnesota, summer home ranges averaged 92.2–193.7 ha [24]. Our fawn home ranges were most similar in size and composition to 3-month home ranges in two suburban Chicago forest preserves, which averaged 12 and 27 ha [15]. Differences in habitat composition are likely the best explanation for the observed differences in home range sizes. Our study area was >90% woodland and grassland in comparison to the Michigan and South Dakota/Minnesota studies, where the landscape was 52–86% agricultural habitat [17, 18, 24]. The large amount of woodland and grassland in our study area meant that fawns did not have to travel far to find suitable cover, which may be responsible for their smaller home ranges.

Female fawns in our study avoided agricultural habitat in their home ranges. Fawns in Michigan also avoided agriculture and other areas of low cover while selecting for woodland within their home ranges [17]. Fawns in South Dakota avoided agriculture in early summer before transitioning to selection for corn when it matured in late summer [51]. We did not detect a shift in fawn habitat selection for agriculture over time, but our study area had at least 80% permanent cover (i.e., woodland). Transitions in habitat selection as documented by [51] may be more important in areas with little permanent cover.

### Cause of mortality

Disease accounted for 43% of recorded mortalities in our study. In comparison, other Midwestern fawn studies reported little-to-no disease mortality (e.g., 1 in [25], 2 in [18]). EHD was responsible for 56% of all our recorded disease events. EHD is a virus spread by insects that typically occurs in late summer and early fall [52]. Outbreaks of EHD occur sporadically, but can cause significant mortality in local deer populations over a short period of time [53]. We recorded EHD mortality only in 2016, beginning in early August and ending in late September, when sustained frost likely killed the midges responsible for spreading the disease [54]. All other disease mortalities occurred within 2 weeks of capture. These early mortalities caused by enteritis and sepsis are consistent with previous studies that considered disease to primarily be a mortality risk early in a fawn’s life [18, 25]. Our findings indicate outbreaks of sporadic diseases like EHD can be a major source of mortality in older age fawns in some years.

Unlike our study, many studies of fawn mortality, including those in the Midwest, documented predation as the leading cause of mortality [see 16]. For example, studies in Iowa and Minnesota/South Dakota attributed 77% and 80% of mortality respectively to predation [24, 25]. Coyotes are responsible for most fawn predation in the Midwest [25, 42] and are the primary wild predator in our study area [29]. The lower predation in our study relative to other studies might be related to habitat differences. The study area in Minnesota/South Dakota reporting 80% predation-related mortality [25], for instance, was 11% woodland, compared to 80% in our study. One previous study found similar canid predation rates in forested, agricultural, and mixed habitats [16], while another found that coyote predation on fawns was higher at a site with low vegetation density and height compared with another, more vegetated, site [15]. Woodland habitat made up a high proportion (~70%, Table 5) of fawn home ranges in our study, which meant fawns had ready access to permanent cover which may have reduced predation rates. Another possible explanation for the low level of predation documented in this study could, like with our survival estimates, be related to our capture methodology where we may have missed predation-caused mortality soon after birth. In fawn studies in Michigan with capture methods and a predator community similar to ours, 50–70.6% of recorded mortalities occurred after 60 days of age and were attributed primarily to harvest and vehicle collisions while predation-associated mortality was low [17, 18]. They suggested availability of protective ground cover and alternate food sources for coyotes may have reduced the impact of coyote predation on fawns.

Our study of white-tailed deer fawn survival and home range in Iowa updates estimates from several decades ago. Our survival estimates are similar to those from other Midwestern states, except that 7-month survival was lower than expected. Home ranges were smaller than those in other similar studies, possibly as a result of the large amount of permanent cover in our study area. We also documented that disease was a significant source of mortality, which is unusual among studies of fawn survival. This study’s updated information on fawn survival can be used in managing Iowa’s deer herd.
